# Imaging findings in coronavirus infections: SARS-CoV, MERS-CoV, and SARS-CoV-2

**DOI:** 10.1259/bjr.20200515

**Published:** 2020-07-06

**Authors:** Tomas Franquet, Yeon Joo Jeong, Hiu Yin Sonia Lam, Ho Yuen Frank Wong, Yeun-Chung Chang, Myung Jin Chung, Kyung Soo Lee

**Affiliations:** 1 Department of Radiology, Hospital de Sant Pau. Universidad Autónoma de Barcelona, Barcelona, Spain; 2 Department of Radiology and Biomedical Research Institute, Pusan National University Hospital, Busan, Korea; 3 Department of Radiology, Queen Mary Hospital, Hong Kong, China; 4 Department of Medical Imaging, National Taiwan University Hospital and Department of Radiology, National Taiwan University College of Medicine, Taipei, Taiwan; 5 Department of Radiology, Samsung Medical Center, Sungkyunkwan University School of Medicine (SKKU-SOM), Seoul, Korea

## Abstract

During the first two decades of the 21st century, there have been three coronavirus infection outbreaks raising global health concerns by severe acute respiratory syndrome coronavirus (SARS-CoV), the Middle East respiratory syndrome coronavirus (MERS-CoV), and the SARS-CoV-2. Although the reported imaging findings of coronavirus infection are variable and non-specific, the most common initial chest radiograph (CXR) and CT findings are ground-glass opacities and consolidation with peripheral predominance and eventually spread to involve both lungs as the disease progresses. These findings can be explained by the immune pathogenesis of coronavirus infection causing diffuse alveolar damage. Although it is insensitive in mild or early coronavirus infection, the CXR remains as the first-line and the most commonly used imaging modality. That is because it is rapid and easily accessible and helpful for monitoring patient progress during treatment. CT is more sensitive to detect early parenchymal lung abnormalities and disease progression, and can provide an alternative diagnosis. In this pictorial review, various coronavirus infection cases are presented to provide imaging spectrums of coronavirus infection and present differences in imaging among them or from other viral infections, and to discuss the role of imaging in viral infection outbreaks.

## Introduction

**Figure 1.**
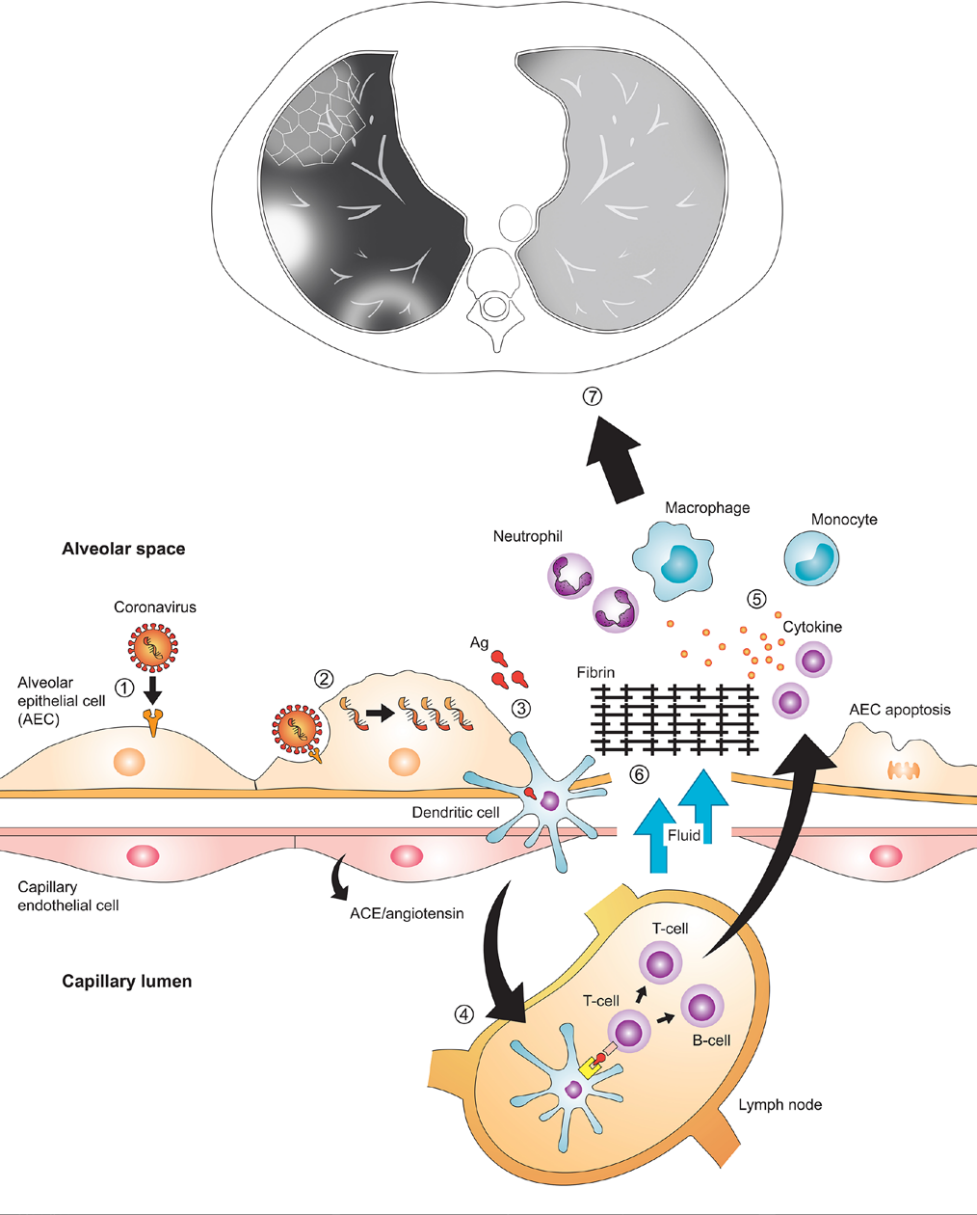
Immune-pathogenesis of coronavirus infection. To enter into host cells, envelope spike glycoprotein of coronavirus binds to the cellular receptor of host cells (1). After the virus enters the cells, the viral RNA genome is released and replicated (2). While the virus enters the cells, its antigen is presented to antigen-presenting cell such as dendritic cell or macrophage (3), which migrates to regional lymph nodes and stimulates the body’s humoral and cellular immunity by virus-specific B and T cells (4). Virus-specific B and T cells produce antibodies and release numerous cytokines. Cytokines induce the recruitment of neutrophils and monocyte-derived alveolar macrophage, and these amplify the inflammatory response (5). The exuberant inflammatory response can disrupt the alveolar-capillary barrier, resulting in alveolar flooding and intra-alveolar fibrin accumulation (6). Cytokine storm, which is an uncontrolled systemic inflammatory response resulting from the release of a large amount of pro-inflammatory cytokines, causes acute respiratory distress syndrome and multiorgan failure. These immune responses result in diffuse alveolar damage or organizing pneumonia, which are manifested as ground-glass opacity, consolidation, crazy paving appearance, and reversed halo sign with peripheral predominance on CT (7).

In late December 2019, the 2019 novel coronavirus pneumonia caused by severe acute respiratory syndrome coronavirus 2 (SARS-CoV-2) occurred in Wuhan, China, and has spread quickly worldwide. During the first two decades of the 21st century, there have been three coronavirus infection outbreaks raising global health concerns by the SARS-CoV, the Middle East respiratory syndrome coronavirus (MERS-CoV), and the SARS-CoV-2 (the respiratory disease caused by SARS-CoV-2 is now called coronavirus disease-19, COVID-19). Coronaviruses are enveloped viruses with a positive-sense single-stranded RNA genome and have club-shaped surface-spike glycoprotein which plays an important role in binding to receptors on host cells. After the virus enters the cells, its antigen is presented to dendritic cell or macrophage, which stimulates the body’s humoral and cellular immunity (Figure 1). Coronavirus induces lung injury by involving angiotensin converting enzyme, by cell apoptosis induced by specific viral proteins, and by cytokine storm, the uncontrolled systemic inflammatory response resulting from the release of large amounts of pro-inflammatory cytokine and chemokines^1^ (Figure 1).

It has been reported that CT patterns of pulmonary viral infection are related to their pathogenesis and most viral pneumonia patterns exhibit similarity based on viridae.^2^ Therefore, the imaging findings of coronavirus infection caused by SARS-CoV, MERS-CoV, and SARS-CoV-2 closely resemble each other. The purpose of this pictorial review is to provide imaging spectrums of coronavirus infection and present differences in imaging among them or from other viral infections, and discuss the role of imaging in viral infection outbreaks.

### Epidemiology and clinical characteristics of human coronavirus infection

Table 1 presents the epidemiology and clinical characteristics of three outbreaks of coronavirus infection. Although the mortality rate of SARS-CoV-2 infection is the lowest among the three outbreaks, the number of confirmed cases of SARS-CoV-2 infection worldwide has exceeded 6,700,000, which is about 2,700 times that of MERS-CoV infection. Males are more susceptible to MERS-CoV and SARS-CoV-2 infections. Up to half of the patients with coronavirus infection have comorbidities such as diabetes, hypertension, and cardiovascular disease and older age has been reported as an important predictor of increased mortality in coronavirus infection.^3–5^ Clinical and laboratory features of coronavirus infection by SARS-CoV, MERS-CoV, and SARS-CoV-2 have some similarities among them. Fever, cough, and dyspnea are the major symptoms in those admitted to the hospital. Gastrointestinal symptoms such as vomiting or diarrhea are relatively rare in SARS-CoV-2 infection, compared to 20–26% of patients with SARS-CoV or MERS-CoV infection having gastrointestinal symptoms.^3,4^ In terms of laboratory findings, a significant number of patients have lymphocytopenia in coronavirus infection, indicating the consumption of immune cells and resultant impairment of cellular immunity.^3,4^

**Table 1.** Epidemiology and clinical characteristics of human coronavirus infection

Characteristics	SARS-CoV	MERS-CoV	SARS-CoV-2
Epidemiology			
Outbreaks (year)	2002–2004	2012, 2015, 2018	2019–2020
Confirmed cases	8,422	2494	6,799,713*
Death	916	858	397,388*
Mortality rates %	11	34.4	5.8*
Age (years)	44	56	51–56
Sex ratio (male to female)	0.8:1	3.3:1	1.7:1
Underlying diseases %			
Diabetes	24	36–66	11–19
Hypertension	19	31–65	19–30
Cardiovascular disease	10	20–41	4–8
Malignancy	3	2	0.5–1
Symptoms %			
Fever	99–100	40–100	94
Cough	62–100	29–100	79–81
Dyspnea	40–42	48–100	40
Myalgia	45–61	32	15–32
Headache	20–56	13	10–14
Sore throat	9–25	7–83	5–8
Nausea or vomiting	20–35	21	4
Diarrhea	20–32	26	5
Laboratory findings %			
Lymphopenia	65–89	34	40–65
Increased CRP	78	63	52–86
Thrombocytopenia	33–46	36	23–42
LDH elevation	56–87	49	67–98

CRP, C-reactive protein; LDH, lactate dehydrogenase; MERS-CoV, Middle East respiratory syndrome coronavirus; SARS-CoV, severe acute respiratory syndrome coronavirus; SARS-CoV-2, severe acute respiratory syndrome coronavirus 2.

a Situation on June 07, 2020

### Imaging findings of coronavirus infection

### Initial and follow-up chest radiographs (CXR) findings

**Figure 2.**
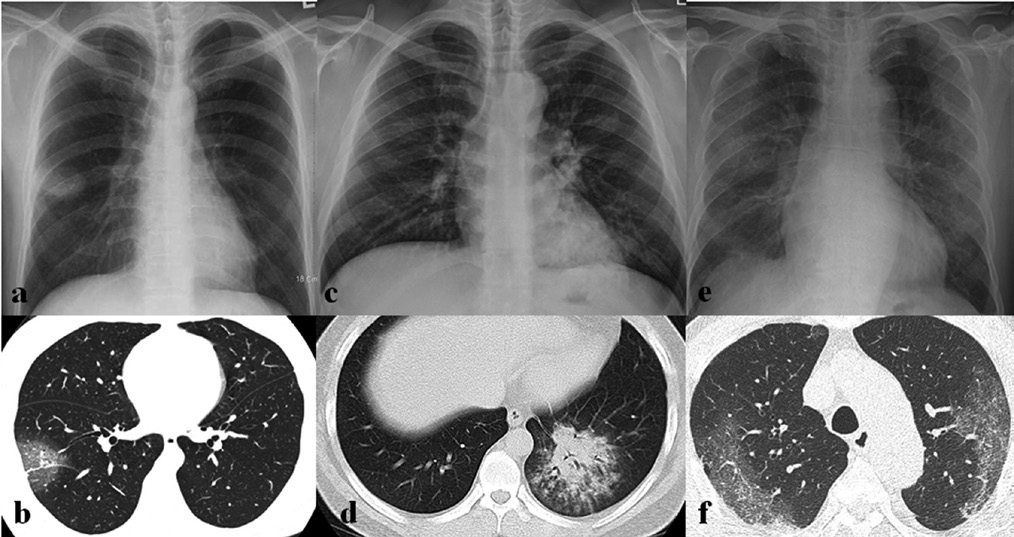
Initial imaging presentation of coronavirus infection. (a) Initial chest radiograph (CXR) obtained from a 52-year-old male patient with SARS-CoV infection shows a focal nodular opacity in the peripheral area of right middle lung zone. (b) Initial CT scan obtained from a 47-year-old female patient with SARS-CoV infection shows focal area of ground-glass opacity (GGO) with crazy paving appearance in the peripheral portion of right lower lobe. (c, d) Initial CXR and CT scan obtained from a 36-year-old male patient with MERS-CoV infection show multifocal patchy consolidation in left middle to lower lung zones on CXR (c) and focal airspace consolidation with air-bronchogram, ill-defined nodules, and peripheral GGOs on CT scan (d). (e, f) Initial CXR and CT scan obtained from a 73-year-old male patient with SARS-CoV-2 infection show bilateral multifocal GGOs on CXR (e) and patchy areas of GGO with crazy paving appearance with peripheral predominance on CT scan (f).

**Figure 3.**
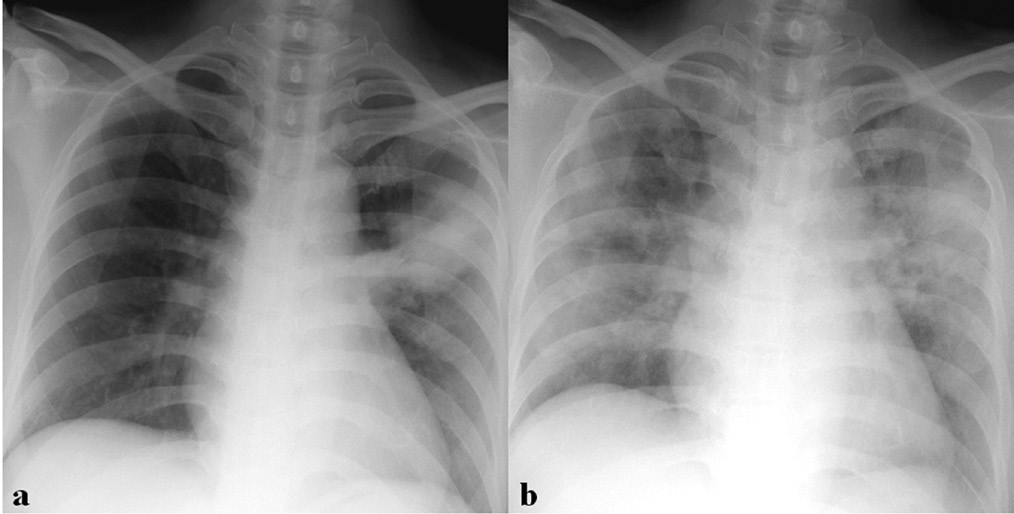
A 36-year-old male patient with SARS-CoV infection. (a) Initial chest radiograph (CXR) shows focal nodular consolidation in left upper lung zone. (b) Follow-up CXR obtained 6 days after (a) shows rapid progression with bilateral diffuse consolidation and GGOs.

**Figure 4.**
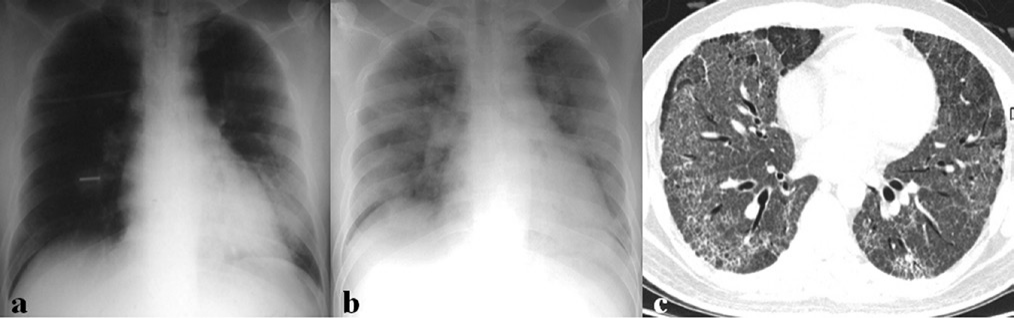
A 31-year-old male with SARS-CoV infection. (a) Initial chest radiograph (CXR) obtained on the day of admission shows unilateral lobar consolidation in left lung. (b) Follow-up CXR obtained 4 days after (a) shows rapid progression with bilateral diffuse ground-glass opacities (GGOs) and consolidation. (c) Follow-up chest CT obtained 40 days after admission shows bilateral diffuse GGOs with superimposed intralobular interstitial thickenings and reticulation, suggesting pulmonary fibrosis.

**Figure 5.**
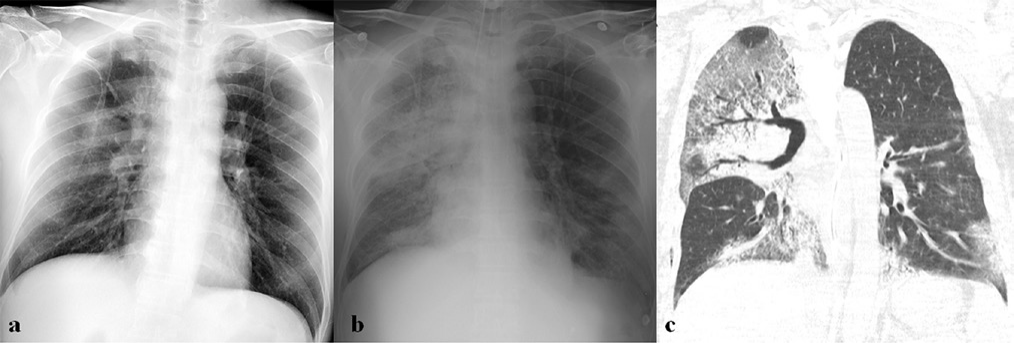
A 60-year-old male patient with MERS-CoV infection. (a) Chest radiograph (CXR) obtained on 8 days after exposure shows consolidation and GGOs in right upper lung zone. (b) Follow-up CXR obtained 18 days after exposure shows multifocal consolidations in both lungs. (c) Follow-up chest CT obtained on the same day with (b) shows bilateral, multifocal, mixed consolidation and GGOs with crazy paving appearance, suggesting organizing pneumonia.

**Figure 6.**
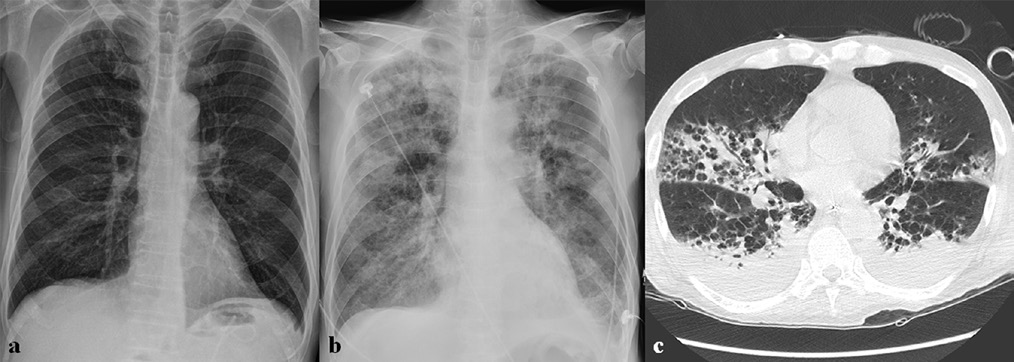
A 68-year-old male with MERS-CoV infection. (a) Initial chest radiograph (CXR) obtained 1 day after symptom onset shows ill-defined patchy ground-glass opacities (GGOs) in both middle to lower lung zones. (b) Follow-up CXR obtained 14 days after symptom onset shows rapid progression with bilateral diffuse GGOs and consolidation in both lungs. Also note blunting of left costophrenic angle, suggesting pleural effusion. (c) Follow-up chest CT obtained 18 days after symptom onset shows bilateral consolidation and GGOs with bilateral pleural effusion. He died of respiratory failure.

**Figure 7.**
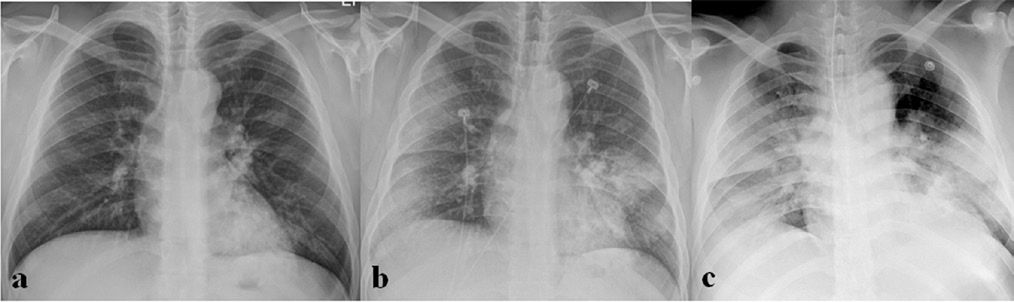
A 36-year-old male patient with MERS-CoV infection. He had a contact history of MERS patients and suffered from fever. (a) Initial chest radiograph (CXR) shows multifocal patchy consolidation in left middle to lower lung zones. (b) Follow-up CXR obtained 15 days after (a) shows increased extents of multifocal patchy consolidation in left middle to lower lung zones and new appearance of diffuse ground-glass opacities and consolidation in the peripheral portion of right lung. (c) Follow-up CXR obtained 18 days after (a) shows bilateral diffuse consolidation, suggesting acute respiratory distress syndrome.

Table 2 summarizes imaging findings of coronavirus infection. Approximately 15%–20% of patients with coronavirus infection have normal initial CXR.^6–8^ The most common initial CXR findings of coronavirus infection are peripheral focal or multifocal airspace consolidation, ground-glass opacities (GGO), or both^6–8^ (Figures 2–5). However, initial imaging in SARS-CoV (Figures 2–4) and MERS-CoV infections (Figures 2 and 5) usually shows unilateral and middle-to-lower lung zone involvement of parenchymal lesions, whereas bilateral lung involvements with lower lung zone or no zonal predominance are more common in SARS-CoV-2 infection^6–8^ (Figure 2). Cavity and nodular opacity are rare in coronavirus infection. Pleural effusion is rare in SARS-CoV-2 infection, but it occurs approximately 15 and 30% of SARS-CoV and MERS-CoV infections during first week of infection^6,7^ (Figure 6). As the disease progresses, parenchymal abnormalities eventually spread to involve the lungs bilaterally in coronavirus infection (Figures 3–7).

**Table 2.** Imaging findings of coronavirus infection

Characteristics	SARS-CoV	MERS-CoV	SARS-CoV-2
**Chest Radiograph**			
**Initial imaging**			
Normal finding %	20	17	31
Parenchymal abnormalities			
GGO	+	+++	+++
Consolidation	+++	+	++
Nodule	-/+	-/+	-
Cavity	-	-/+	-
Pleural effusion	+	++	-/+
Pneumothorax	-	+	-
Distribution			
Transverse	Peripheral	Peripheral	Peripheral
Longitudinal	Lower lung	Middle & lower lung	Lower lung/random
Laterality	Unilateral	Unilateral	Bilateral
**Follow-up imaging with progression**	Entire bilateral lung	Entire bilateral lung	Entire bilateral lung
**Prognostic determinant**	CXR score	CXR score, pleural effusion, pneumothorax	CXR score
**Chest tomography**			
**Initial imaging**			
Parenchymal abnormalities			
GGO	+++	+++	++++
Consolidation	++	++	++
Mixed GGO & consolidation	++	++	++
Intralobular interstitial thickening	++	+	++
Interlobular septal thickening	++	++	++
Crazy paving appearance	++	+	+
Reticulation	-	-	-
Traction bronchiectasis	-	-	-
Large nodule	-	-	-
Centrilobular nodule	-	-/+	-
Cavity	-	-	-
Pleural effusion	+	++	-
Pneumothorax	-	-	-
Hilar or mediastinal LN enlargement	-	-	-
Distribution	Peripheral, lower lung	Peripheral, lower lung	Peripheral, random
Laterality	Unilateral	Unilateral	Bilateral
**Follow-up imaging after 4 weeks**			
Predominant findings	Signs of fibrosis	Signs of fibrosis	Signs of fibrosis
**Prognostic determinant**	CT score	CT score, pleural effusion	CT score

SARS-CoV = severe acute respiratory syndrome coronavirus, MERS-CoV = Middle East respiratory syndrome coronavirus, SARS-CoV-2=severe acute respiratory syndrome coronavirus 2,

a extent of each parenchymal abnormality <25%, ++;≥25%,<50%, +++;≥50%,<75%, ++++;≥75%, GGO = ground-glass opacity, CXR = chest radiograph, LN = lymph node

### Initial and follow-up chest CT findings

**Figure 8.**
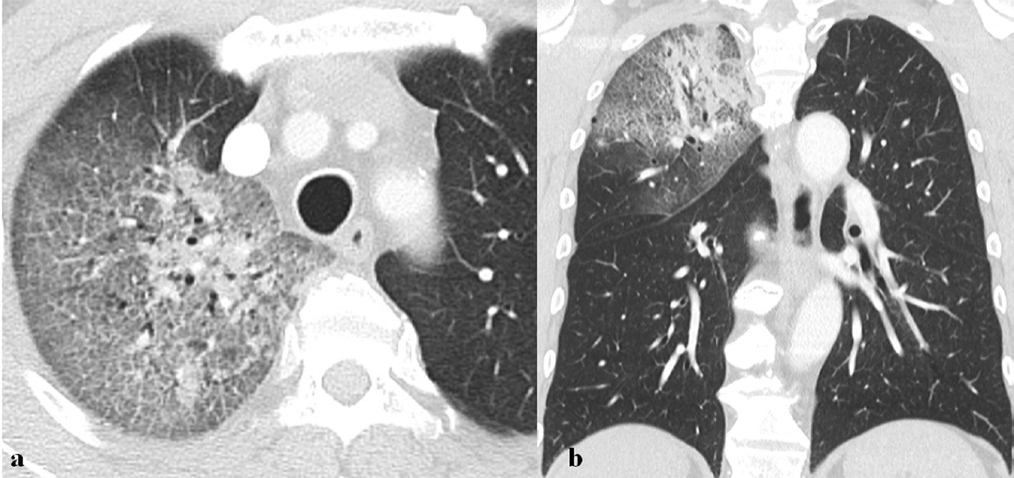
A 68-year-old male patient with MERS-CoV infection. He had a history of visiting Bahrain and suffered from fever, myalgia, and dry cough. (a, b) Axial and coronal images of chest CT scan obtained 4 days after symptom onset show mixed consolidation and ground-glass opacity with the crazy paving appearance in right upper lobe.

**Figure 9.**
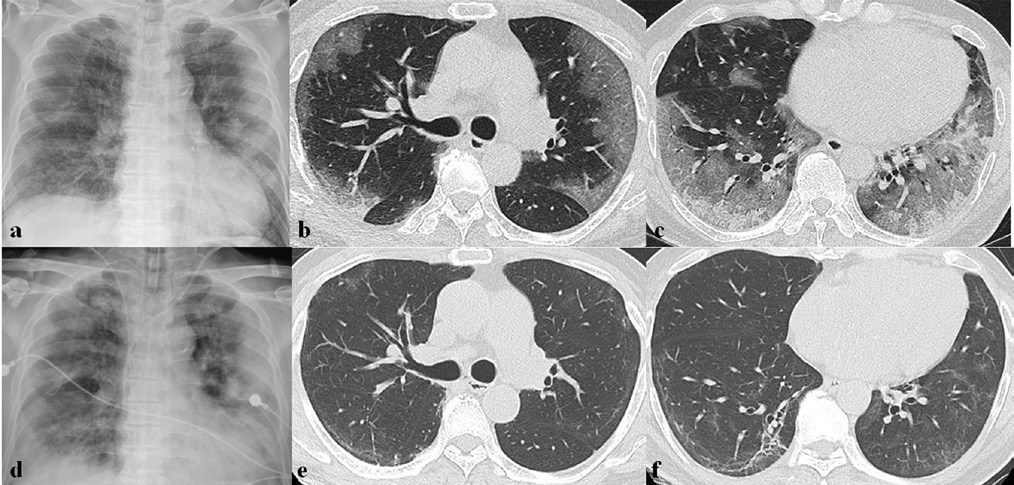
A 64-year-old male patient with SARS-CoV-2 infection. (a) Initial chest radiograph (CXR) obtained 7 days after symptom onset shows bilateral multifocal patchy ground-glass opacities (GGOs) with peripheral predominance. (b, c) Chest CT scans obtained on the same day with (a) show multifocal patchy and diffuse GGOs with the crazy paving appearance and mixed GGO and consolidation in both lungs. (d) Follow-up CXR after 2 days of (a) shows rapid progression with bilateral diffuse consolidations and GGOs. (e, f) Follow-up chest CT scans obtained 1 month later show markedly decreased extent of GGO with remnant subpleural line and traction bronchiectasis, suggesting pulmonary fibrosis.

**Figure 10.**
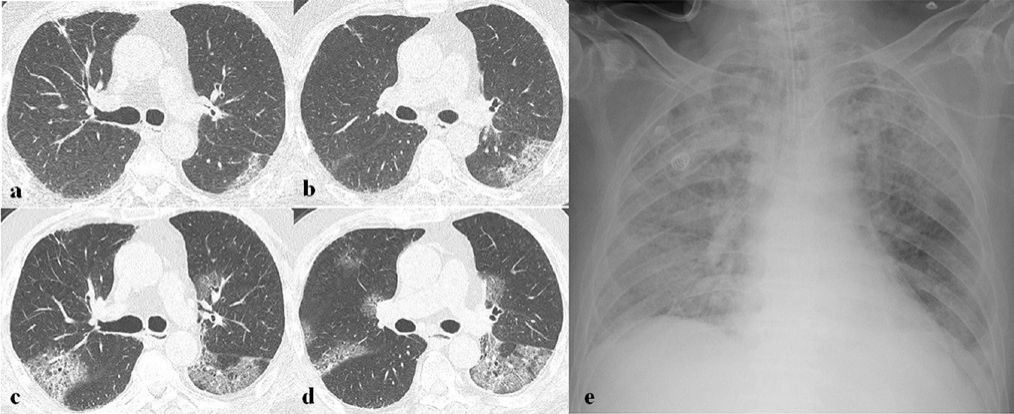
A 78-year-old male patient with SARS-CoV-2 infection. He had a history of exposure to an infected patient and had fever and myalgia for 1 day. (a, b) Initial chest CT scans obtained 2 days after symptom onset show focal subpleural ground-glass opacity (GGO) in left lower lobe. (c, d) Follow-up CT scans obtained 7 days after symptom onset show increased extent of GGO in left lower lobe and new appearance of multifocal subpleural patchy GGOs with the crazy paving appearance in both lungs. (e) Follow-up chest radiograph obtained 15 days after symptom onset shows bilateral diffuse GGOs and consolidation in both lungs, suggesting diffuse alveolar damage. He died of respiratory failure.

**Figure 11.**
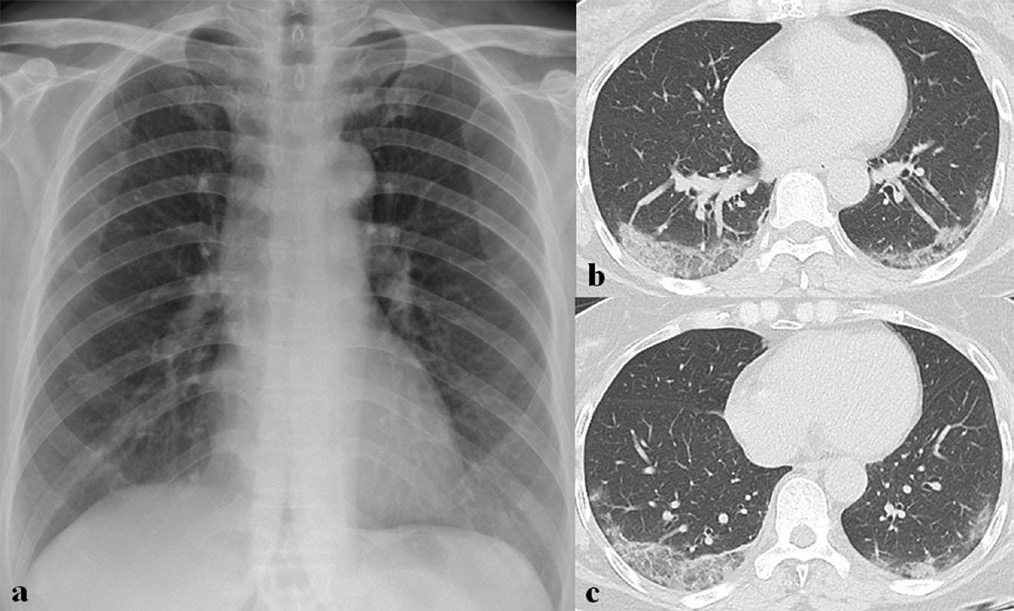
A 64-year-old female patient with SARS-CoV-2 infection. (a) Initial chest radiograph shows faint ground-glass opacities (GGOs) in both lower lung zones. (b, c) Follow-up CT scans obtained 14 days after symptom onset show mixed GGOs and consolidation in both lower lung lobes.

**Figure 12.**
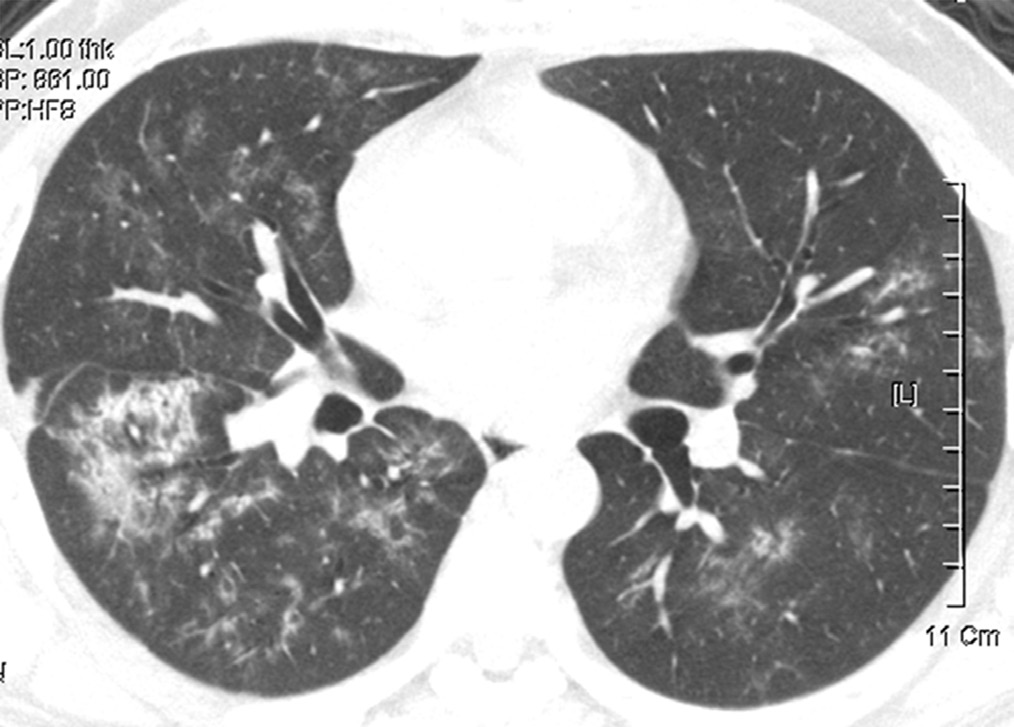
A 43-year-old male with SARS-CoV infection. Chest CT obtained 25 days after symptom onset shows bilateral diffuse ill-defined centrilobular and ground-glass opacity nodules in both lungs. Also, note focal consolidation in right lower lobe.

**Figure 13.**
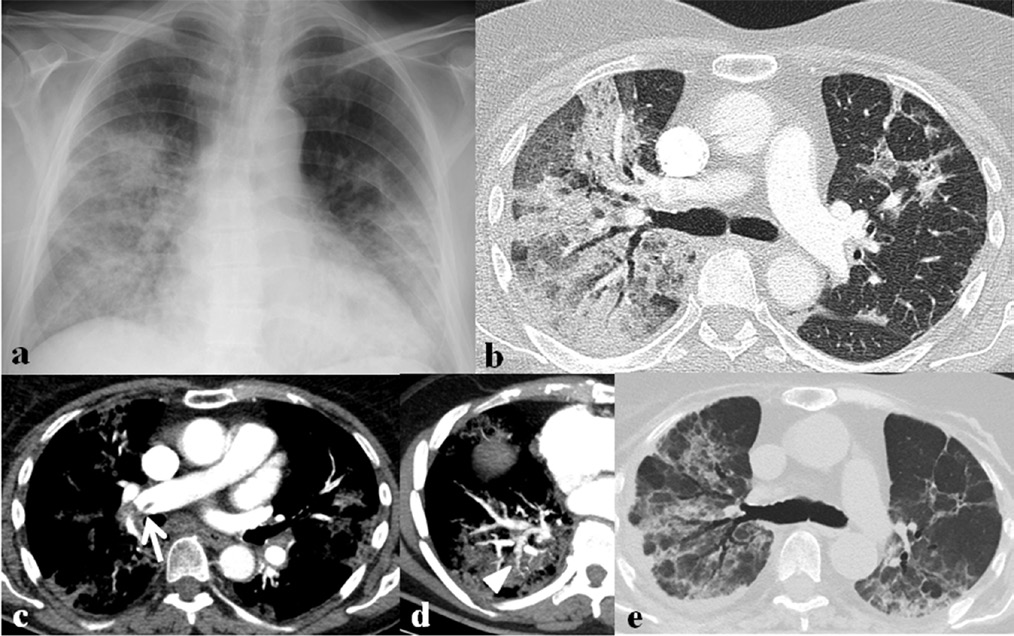
A 78-year-old female patient with SARS-CoV-2 infection. (a) Initial chest radiograph shows bilateral diffuse consolidation in both lungs. (b) Lung window image of contrast-enhanced chest CT scan obtained on the same day with (a) shows mixed ground-glass opacities (GGOs) and consolidation in both lungs. (c, d) Mediastinal window images of contrast-enhanced chest CT scan obtained on the same day with (a) show thromboembolism (arrow) involving right interlobar pulmonary artery and irregular-walled vessels (arrowhead) toward consolidation in right lower lobe. (e) Follow-up chest CT scan after 1 month of (a) shows decreased extent of mixed GGOs and consolidation with remnant multifocal patchy GGOs, parenchymal bands, traction bronchiectasis, and irregular interface, suggesting pulmonary fibrosis.

**Figure 14.**
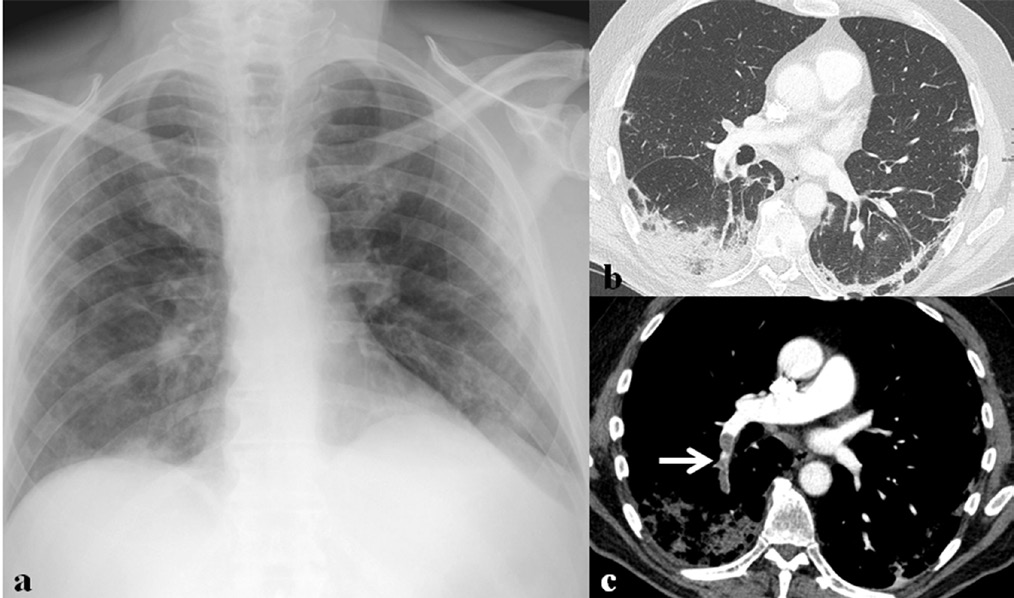
A 71-year-old male patient with SARS-CoV-2 infection. (a) Initial chest radiograph shows bilateral peripheral ground-glass opacities (GGOs) and multifocal patchy consolidation in both lungs. (b) Lung window image of contrast-enhanced chest CT scan obtained on the same day with (a) shows consolidation and patchy GGOs in the subpleural areas of both lower lobes. (c) Mediastinal window image of contrast-enhanced chest CT scan obtained on the same day with (a) shows thromboembolism (arrow) involving right lower lobar pulmonary artery.

Similar to the CXR findings of coronavirus infection, the CT findings include GGO with or without interlobular septal thickening, consolidation, or a combination of both; these are the most common findings during the first two weeks of coronavirus infection^9–11^ (Figures 2 and 8–10). In SARS-CoV infection, reticulation is evident after the second week of infection and persists in half of the patients after 4 weeks^9^ (Figure 4). In MERS-CoV infection, crazy paving abnormalities and organizing pneumonia (OP) are seen during the second and third weeks of infection^10^ (Figure 5). In SARS-CoV-2 infection, GGO (with or without crazy paving appearance) and consolidation show a decrease in extents, whereas a mixed pattern of GGO and consolidation demonstrates an increase after second week of infection^11^ (Figure 11). Cavity, centrilobular nodules (Figure 12), mediastinal, or hilar lymph node enlargement are rarely seen in coronavirus infection. Pulmonary vascular enlargements in areas of lung opacity can be seen in more than half of the patients with SARS-CoV-2 infection^12^ and venous or arterial thromboembolic disease may be complicated in about 30% of critically ill patients with SARS-CoV-2 infection due to excessive vascular inflammation or diffuse intravascular coagulation^13^ (Figures 13 and 14).

**Figure 15.**
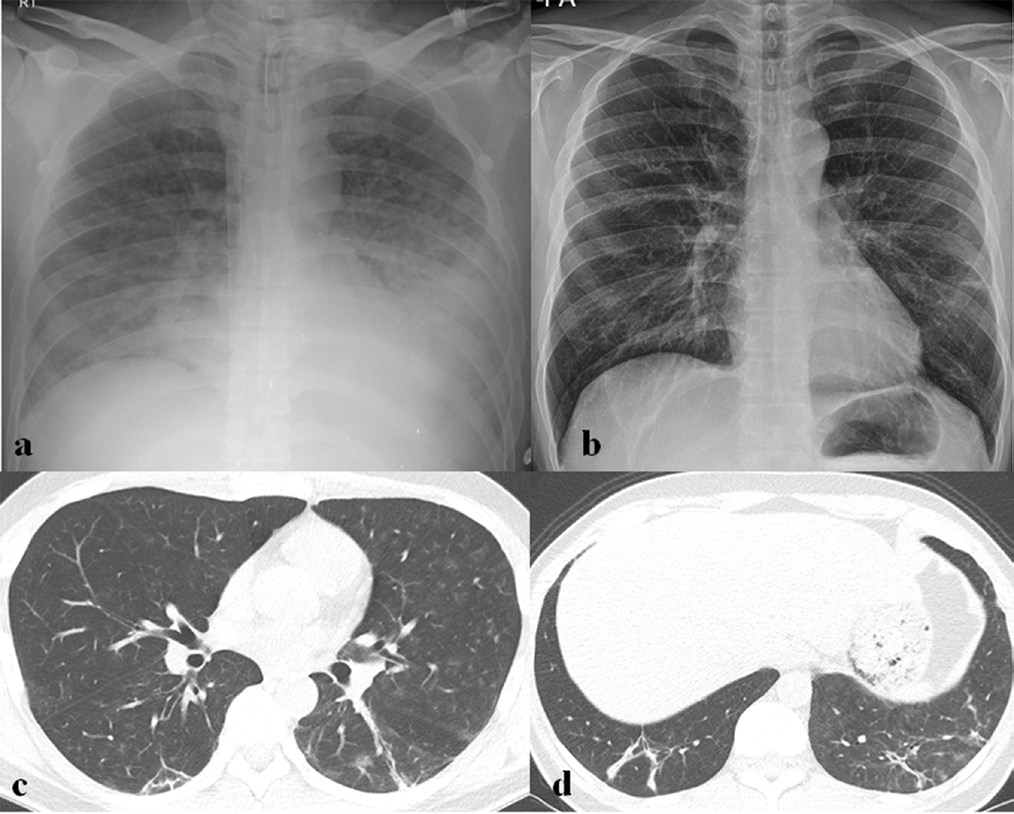
A 32-year-old male with MERS-CoV infection. (a) Chest radiograph (CXR) obtained on 15 days after symptom onset shows consolidation and ground-glass opacities in both middle to lower lung zones. (b) Follow-up CXR obtained 50 days after symptom onset shows linear and patchy opacities in both lower lung zones, which may represent fibrosing sequelae. (c, d) Follow-up chest CT scans obtained on the same day with (b) show parenchymal bands, linear opacities, and traction bronchiectasis in both lower lobes, suggesting remnant fibrosis.

The location and distribution of parenchymal abnormalities and disease progression patterns are similar to those seen on CXRs.^9–11,14^ During the early stage of the disease, parenchymal abnormalities are usually located in the peripheral lung; from there, they eventually spread to involve both lungs when the disease progresses (Figure 10). Parenchymal abnormalities usually have a peripheral and basilar predilection in MERS-CoV infection^10^ (Figures 2 and 5), whereas with peripheral or both peripheral and central predilection bilateral multilobe lesions are seen in SARS-CoV-2 infection^11,14^ (Figures 2, 9–11, 13 and 14). In the chronic stage of coronavirus infection, variable-sized GGO with interlobular septal and intralobular interstitial thickenings are the most common findings. Signs of fibrosis such as parenchymal bands, reticulation, traction bronchiectasis, irregular interface signs are also usually seen^9–11^ (Figures 4, 9, 13 and 15). In SARS-CoV infection, GGO and interstitial opacity in the convalescent phase usually resolve over time but air trapping may persist.^15^

### Prognostic determinants

Several studies have been conducted to investigate the correlation between imaging findings and clinical outcomes. Higher CT and CXR scores (percentage of lungs or the number of zones involved with opacification), especially in patients with old age and comorbid lung illness, could be used as fatal prognostic indicators in SARS-CoV infection.^5^ In MERS-CoV infection, a greater extent of parenchymal abnormalities, pleural effusion, and pneumothorax was associated with poor prognosis and short-term mortality (Figure 6).^10,16^ Although imaging features that help determine the prognosis of SARS-CoV-2 infection are not yet fully understood, older age and progressive consolidation with a greater extent on imaging might suggest poorer prognosis (Figure 10).

### Differential diagnosis

Coronavirus infection is sometimes difficult to distinguish from other viral infections, bacterial pneumonia, or OP associated with other causes. Viral pneumonia in an immunocompetent patient can be divided into bronchopneumonia, OP, and diffuse alveolar damage (DAD) pattern. OP and DAD pattern are common features of coronavirus pneumonia and can be seen during the disease course or in advanced disease. Viral pneumonia showing OP or DAD patterns include influenza virus, Hantavirus, adenovirus, and varicella-zoster virus pneumonia. Consolidation and GGO with subpleural or peribronchovascular predominance are typical imaging findings of OP. Therefore, cryptogenic OP and OP associated with infection, drug, and collagen vascular disease should be considered to be differentiated from coronavirus infection.

It is difficult to differentiate coronavirus infection from other diseases by imaging alone; therefore, clinical manifestation, contact history, and laboratory tests should also be considered to make the final diagnosis.

### Role of imaging in coronavirus infection

The main role of imaging in viral infection outbreak is to identify the presence of pneumonia, provide differential diagnosis, and monitor changes of pneumonia with treatment. When deciding which imaging modality to use for establishing a diagnosis or for guiding management in viral infection outbreak, there are several factors to consider; patient’s clinical severity, radiation hazard to the patient, accessibility of imaging modalities, possibility of virus transmission to uninfected healthcare workers and other patients, and community infection status.^17^

Although it is insensitive in mild or early coronavirus infection, CXR remains the first-line and the most commonly used imaging modality because it is rapid and easily accessible. It is also helpful for monitoring patient progress during treatment. CT is more sensitive to detect early parenchymal lung abnormalities and disease progression, and can provide an alternative diagnosis. However, CT lacks equipment portability with imaging performed within an infected patient’s isolation room and has a risk of virus transmission along the transport route to a CT scanner and within the CT room. CT can be used when the patient has symptoms but the CXR is normal or there are only questionable abnormalities on CXR. CT is also indicated in patients with functional impairment and/or hypoxemia after recovery from coronavirus infection. Contrast-enhanced CT may be indicated in patients with signs of thrombotic complication. The appropriate use of imaging in each clinical situation should be considered on this basis.^17^

## Conclusions

Although imaging findings of coronavirus infection are non-specific and have significant overlap among those of SARS-CoV, MERS-CoV, and SARS-CoV-2 infections, they have several important differences. The most common initial CXR and CT findings are GGO and consolidation with peripheral predominance and these lesions eventually spread to involve both lungs as the disease progresses and pulmonary fibrosis may develop after long-term follow-up. Imaging features that help determine the clinical outcome or prognosis of coronavirus infection are the extents of parenchymal abnormalities. The greater extents of parenchymal abnormalities, especially in patients with old age or comorbidity, might suggest poorer prognosis in coronavirus infection, necessitating intensive care unit management, or predicting oncoming succumbing to death.
